# Medical College Notes

**Published:** 1891-09

**Authors:** 


### Medical College Notes.

THE Medical College year is about to commence, and before another issue of the JOURNAL the two schools in this city will be in full blast. The Buffalo University will open on Monday, September

28, 1891, and announces a three years' course of graded instruction, in compliance with recent laws, and in keeping with the general advance of public sentiment on the subject of improved medical education. The Niagara University will open its regular collegiate year on Monday, September 21, 1891, with an introductory address by Prof. Herman Mynter, M. D. This school has always required three years of instruction, and was organized on the graded plan. It now contemplates advancing to four years of graded instruction as a requirement for its degree.

We have given in our issues for June and July the recent changes in the faculties of these two schools.

---

The medical department of Tulane University was made, June 13th, the recipient of a generous donation from Mrs. Richardson, wife of that eminent physician and dean of the college, Dr. T. G. Richardson, of $100,000. The entire donation is intended to be used in erecting a new college on Canal street, between Vilere and Robertson, the site for which was bought a few days ago for $35,000 by the Educational Board.

The faculty of the medical department of the university has selected Dr. Edmund Souchon, Professor of Anatomy and Clinical Surgery, as its representative in the selection of the proper sort of building for the purpose intended. Dr. Souchon will soon leave for the North and East to examine various colleges to guide him in the selection of a building that will be best suited to the wants of the local institution.—*Medical Bulletin.*

---

Reorganization of the Chicago College of Physicians and Surgeons.— The coming collegiate year will be marked by a number of radical changes in the above institution; several gentlemen have resigned and a number of new professors have been elected. Among the new men are Dr. Bayard Holmes, surgical pathology; Dr. Boerne Bettman, ophthalmology; Dr. James A. Lydston, professor of chemistry and lecturer on ophthalmology and otology; Dr. Weller Van Hook, general pathology; Dr. E. E. Babcock, clinical medicine and diseases of the chest; Dr. G. Frank Lydston, surgical diseases of the genito-urinary organs and venereal diseases.

The college already has an excellent reputation, and the elements of strength which have been added to the faculty will undoubtedly contribute to the usefulness and renown of the institution. The increased confidence of the alumni of the institution augers well for its future prosperity.—*Western Medical Reporter.*

---

At the Medico-Chirurgical College of Philadelphia, Prof. E. Laplace has been elected to the chair of Surgery; J. M. Anders, to that of Practice of Medicine; and E. E. Montgomery, to that of Obstetrics. Thus, each of the vacancies in the great didactic chairs has been filled by men already members of the faculty. Prof. Geo. E. Stubbs will probably resign the chair of Surgical Pathology, leaving only Prof. Gerhard to represent the faculty that created this college ten years ago.—*Medical Bulletin.*

---

The announcement of the Long Island College Hospital for 1891 contains the following items of interest: The regular course of lectures will hereafter be six months in duration. Three courses of lectures will hereafter be required for graduation. Joshua M. Van-Cott, Jr., M. D., has been appointed Professor of Histology and Pathological Anatomy, *vice* Frank Ferguson, M. D., who has resigned.

